# Differential Expression of *fimH*, *ihf*, *upaB*, and *upaH* Genes in Biofilms- and Suspension-Grown Bacteria From Samples of Different Uropathogenic Strains of *Escherichia coli*

**DOI:** 10.1155/ijm/5235071

**Published:** 2024-12-12

**Authors:** Esmeralda Rodríguez-Miranda, María de Lourdes Reyes-Escogido, Viridiana Olmedo-Ramírez, Octavio Jiménez-Garza, Sergio López-Briones, Marco Antonio Hernández-Luna

**Affiliations:** ^1^Translational Biomedicine Laboratory, Department of Medicine and Nutrition, Health Sciences Division, University of Guanajuato, León, Guanajuato, Mexico; ^2^Metabolism Laboratory, Department of Medicine and Nutrition, Health Sciences Division, University of Guanajuato, León, Guanajuato, Mexico; ^3^Clinic Laboratory, Silao General Hospital, Ministry of Health of the State of Guanajuato, Silao, Guanajuato, Mexico; ^4^Health Sciences Institute, Autonomous University of Hidalgo State, Pachuca, Hidalgo, Mexico

**Keywords:** biofilm, urinary tract infections, uropathogenic *Escherichia coli*

## Abstract

Uropathogenic *Escherichia coli* (UPEC) strains are the main bacteria that cause urinary tract infections (UTIs). UPEC are a significant public health hazard due to their high proliferation, antibiotic resistance, and infection recurrence. The ability to form biofilms is a mechanism of antibiotic resistance, which requires the expression of different genes such as *fimH*, *ihf*, *upaB*, and *upaH*. Despite the relevance of biofilm formation in bacterial pathogenicity, differences in the expression level of these genes among bacterial growth conditions have been little studied. Here, we have characterized the expression of *fimH*, *ihf*, *upaB*, and *upaH* genes in biofilms and suspension-grown bacteria of different *E. coli* strains. These included the UPEC CFT073, the multidrug-resistant strain CDC-AR-0346, and clinical isolates obtained from UTI patients. The expression of *fimH*, *ihf*, *upaB*, and *upaH* was markedly heterogeneous in clinical isolates, both in terms of transcript levels and response to suspension or biofilm conditions. That expression pattern was distinct from the one in UPEC CFT073, where *upaB* and *upaH* were upregulated and *ihf* and *fimH* were slightly downregulated in biofilm. In conclusion, the data presented here show that the pattern of biofilm-associated genes in the clinical isolates from UTI patients is not fully related to the reference strain of UPEC CFT073. However, analysis of a larger number of samples is required.

## 1. Introduction

Urinary tract infections (UTIs) are highly frequent and recurrent in humans [1]. The main pathogenic microorganism causing UTI is uropathogenic *Escherichia coli* (UPEC), which has been detected in 80%–90% of UTIs [2]. In addition UPEC can induce cystitis and pyelonephritis [3]. Due to recurrence of UTIs and resistance to antibiotic treatment, UPEC represent a major public health hazard, and contribute to the soaring cost of healthcare worldwide [4, 5].

Bacteria have developed resistance to antibiotics through different mechanisms, including biofilm formation. Biofilm-forming bacteria have been associated with severe UTIs such as catheter-associated UTIs (CAUTI) in the hospital environment [6, 7]. Bacterial biofilms are complex structures composed of polysaccharides, lipids, extracellular DNA (eDNA), and proteins [8, 9]. Biofilm formation occurs in response to environmental stress, such as low nutrient levels, changes in salt and oxygen concentration, and pH [9]. Several stages of biofilm formation have been described, including adhesion of bacteria to the solid surface, cell-to-cell adhesion, maturation, and bacterial aggregation and organization. This allows bacterial self-immobilization on the extracellular polymeric matrix, adopting a multicellular biology. These phenomena are regulated by the expression of different genes [10]. In this regard, biofilm formation by *E. coli* requires activation of genes that promote host cell adhesion [11] and bacterial aggregation [12]. These genes include Type 1 fimbriae, a specific D-mannose adhesin (*fimH*) [13], Integration Host Factor (*ihf*) [14], and autotransporters (ATs) encoded by genes such as *upaB* and *upaH* [15, 16]. Interestingly, in all cases a decrease in biofilm formation has been detected when these genes were deleted in UPEC strains. However, the conditions that regulate the expression of *fimH*, *ihf*, *upaB*, and *upaH* genes are poorly understood.

Therefore, the aim of this study was to determine the expression of *fimH*, *ihf*, *upaB*, and *upaH* in different *E. coli* strains grown in suspension or in biofilm.

## 2. Materials and Methods

### 2.1. Bacterial Strain Samples

Different clinical isolates were used in this study. The UPEC *E. coli* strain CFT073 (ATCC 700,928, Manassas, VA, USA) and the multidrug-resistant *E. coli* strain CDC-AR-0346 (Microbiologic Saint Cloud, MN, USA) were used as reference. Twelve clinical isolates of *E. coli* were obtained from UTI patients attending a local hospital (Silao, Guanajuato, Mexico). Briefly, urine samples were plated on MacConkey agar (MCD LAB, Tultitlan, Edo Mex, México). Lactose-positive colonies were subjected to identification for *E. coli*, and then antibiogram was performed on an automated BD Phoenix M50 with BD Phoenix UNMIC/ID-407 panel (Becton Dickinson Franklin Lakes, NJ, USA). All *E. coli* strains were isolated and classified according to the clinical record number (i.e., “Uro” followed by an ID number: Uro547).

### 2.2. Bacterial Growth Curves

All bacterial samples were grown overnight at 37°C in Luria-Bertani (LB) broth (MCD LAB, Tultitlán, Edo Mex, México). The next day, a 1:1000 dilution of the overnight culture was performed in fresh LB medium and grown at 37°C. Bacterial growth was determined by spectrophotometry at 600 nm every 30 min for 360 min.

### 2.3. Plasmid Detection

Plasmid isolation from all bacterial samples was performed by the alkaline lysis extraction method [17]. Isolated plasmids were subjected to electrophoresis on 1% agarose gels (Invitrogen, Waltham, MA, USA) for 40 min at 95 V. Agarose gels were stained with SYBR Safe (Invitrogen, Waltham, MA, USA) according to the manufacturer's instructions, and visualized on iBright CL1500 imaging system (Thermo Fisher Scientific Waltham, MA, USA).

### 2.4. Biofilm Formation and Quantification

Biofilm was determined in overnight cultures of all bacterial samples. Bacteria were cultured in 1 mL of LB medium in sterile Falcon 12 × 75 mm polystyrene tube, (Corning, Glendel, AZ, USA), and stained with 0.1% crystal violet (Karal, León, Gto., México). After 24 h of culture at 37°C. The formation of biofilms in the tubes was recorded using an EOS T6 photographic camera (CANON Inc, Melville, NY, USA).

For biofilm quantification, overnight cultures of all samples were performed in 96-well plates, (Corning, Glendel, AZ, USA). After removing the culture medium, the plates were washed 3 times with water and stained with 0.1% crystal violet. The stained biofilm was dissolved with 30% acetic acid (Karal, León, Gto., México) and quantified by spectrophotometry at 550 nm in m200 Infinite Pro Microplate Reader (Tecan Männedorf, Switzerland).

To determine the microscopic structure of the biofilm, all samples were cultured in LB medium for 24 h at 37°C in 8-well slide chambers (SPL, Gyeonggi-do, Korea) then stained with 0.1% crystal violet. The walls of the slide chamber containing the biofilm were cut, analyzed under an optical microscope (Zeiss Oberkochen, Germany) and recorded on an image analyzer (AmScope Irvine, CA, USA).

### 2.5. Detection of *fimH*, *ihf*, *upaB*, and *upaH* by PCR

A genomic DNA extraction kit (Promega Madison, WI, USA) was used to purify bacterial DNA. For the detection of *fimH, ihf, upaB, and upaH* genes, a multiplex PCR was designed using publicly available tools (bioinformatics.org). The PCR primers are listed in Table 1. Amplification conditions were performed according to the manufacturer's instructions for Taq polymerase (PCRBIOSYSTEMS Wayne, PA, USA) for multiplex PCR or Syber Green Master Mix (Golbio Biotechnology St. Louis MO, USA) for RT-PCR, using 250 ng of genomic DNA as template in both cases.

### 2.6. *fimH, ihf, upaB*, and *upaH* Gene Expression Analysis

To determine the mRNA levels of *fimH, ihf, upaB,* and *upaH*, samples of both bacteria grown in suspension and biofilm were obtained. Briefly, all bacterial samples were cultured in 96-well plates at 37°C for 24 h. Suspension cells were then collected from each well and transferred to 1.5 mL microcentrifuge tubes, then each well was washed thoroughly three times with PBS buffer (pH 7.2) to remove the remaining suspension-grown and unattached bacterial cells. Bacteria adhered to the biofilm were directly solubilized with 400 *μ*L Trizol (Invitrogen Waltham, MA, USA) and after thorough pipetting, transferred to 1.5 mL microcentrifuge tubes. Total RNA was purified from both bacteria grown in suspension and biofilm, using Trizol following the manufacturer's instructions. RNA integrity was verified by 1.5% agarose gel electrophoresis running for 40 min at 95 V and quantified by spectrophotometry in m200 Infinite Pro Microplate Reader (Tecan Männedorf, Switzerland). Finally, 2.5 ng of total RNA was used to synthesize cDNA with Accuris qMax cDNA Synthesis Kit, (Accuris Instruments Edison, NJ, USA) following manufacturer's instructions. The cDNA was used for RT-PCR with qPCR Master Mix with SYBR Green (Golbio Biotechnology St. Louis MO, USA) according to the manufacturer's conditions, and primers specific for *the fimH, ihf, upaB, and upaH* genes and the housekeeping control *16S rRNA* subunit in a CFX Opus 96 Real-Time PCR system (Bio-Rad Hercules, CA, USA). Results were analyzed in CFX Maestro software for CFX Real-Time PCR instruments (Bio-rad Hercules, CA, USA). Amplification products were subjected to electrophoresis in 1% agarose gels running for 40 min at 95 V, and detected with an iBright CL1500 imaging system (Thermo Fisher Scientific Waltham, MA, USA).

### 2.7. Statistical Analysis

Statistical analysis was performed with GraphPad Prisma 9.0 software. Differences between groups were determined by analysis of variance (ANOVA) and Student's *t*-test (independent samples). Data were presented as means ± SD. A two-tail *p* < 0.05 was considered statistically significant.

## 3. Results

### 3.1. Antibiotic Resistance of *Escherichia coli* From Clinical Isolates

All samples from clinical isolates were tested by antibiograms to determine the resistance to different antibiotics. We used the *E. coli* strain CFT073 as negative control since it lacks antibiotic resistance.  Table 2 summarizes the family of antibiotics to which the isolated *E. coli* were resistant. For the complete list of antibiotics to which the clinical isolates were resistant, see Supporting Table 1.

*E. coli* from clinical isolates Uro 565 and Uro 649 showed no resistance to any antibiotic. By contrast, all other samples showed variable degrees of antibiotic resistance, with the number of tolerated antibiotics ranging from four to one. Interestingly, tetracycline was the antibiotic to which most clinical isolate *E. coli* were resistant, followed by *β*-lactams. Meanwhile, nitro derivatives and aminoglycosides were the antibiotics to which *E. coli* showed the highest sensitivity except in sample Uro 547, and Uro 566 and Uro 567, respectively.

### 3.2. Detection of Plasmids in *E. coli* From Clinical Isolates

**Figure 1:**
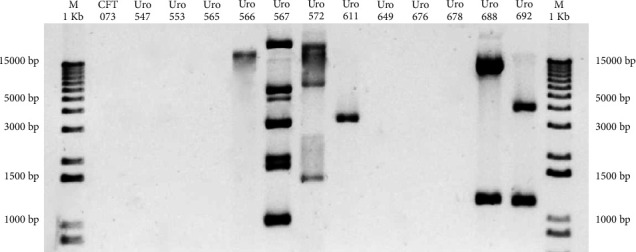
Plasmid detection. Clinical isolates were classified according to clinical history number. Plasmids were isolated from all samples and subjected to electrophoresis on 1% agarose gels, stained with SYBR green, and visualized on the iBright CL1500 imaging system. *E. coli* strain CFT073 was used as a negative control.

Antibiotic resistance genes are often transferred from one bacterium to another via plasmids [18]. Therefore, plasmid detection was carried out in all samples to determine if antibiotic resistance of *E. coli* from clinical isolates depended on acquired plasmids.  Figure 1 shows that plasmids were detected in six out of 12 (50%) clinical isolate *E. coli*. The *E. coli* strain CFT073 was used as a negative control.

### 3.3. Biofilm Formation by *E. coli* From Clinical Isolates

**Figure 2:**
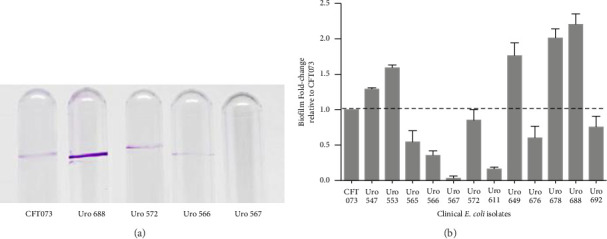
Bacterial capacity to form biofilms. Biofilm formation was determined by crystal violet staining in 5 mL polystyrene tubes and quantified by spectrophotometry assay, as indicated in material and methods. (a) Shows a representative image of biofilm formation in polystyrene tubes of *E. coli* strain CFT073 and clinical isolates Uro 688, Uro 572, Uro 566, and Uro 567. In (b), the graph represents the biofilm fold change relative to *E. coli* strain CFT073, used as a control. Each bar represents the mean ± SD of each sample quantified by triplicate.

**Figure 3:**
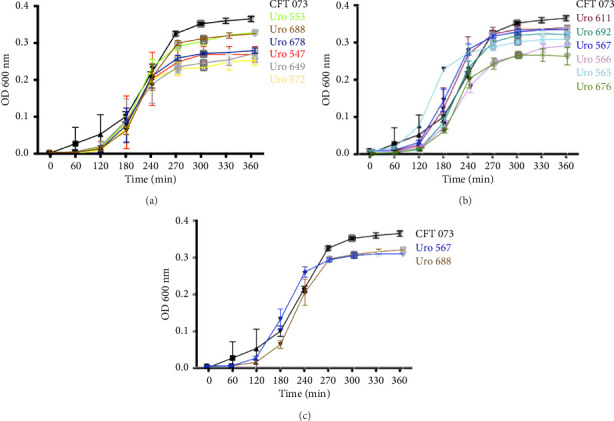
Bacterial growth curves. All bacterial samples were cultured overnight at 37°C. The next day, a fresh culture was grown at 37°C from a 1:1000 dilution of the overnight culture. Bacterial growth was determined by spectrophotometry at 600 nm every 30 min for 360 min. (a) Shows the bacterial growth kinetics of *E. coli* strains from clinical isolates that formed the same or more biofilm than CFT073, while (b) shows the clinical isolates that formed little or no biofilm (b). (c) Shows the comparison of bacterial growth kinetics between Uro 688 (the clinical isolate with greater biofilm formation) and Uro 567 (which did not form biofilms). Each point represents the mean ± SD of each measurement quantified in triplicate.

The ability to form biofilms was confirmed by crystal violet staining in all bacterial clinical isolates. Interestingly, except for the clinical isolate Uro567, biofilm formation was observed in all samples although at different extent.  Figure 2(a) shows a representative image of the tube tests of biofilm formation of *E. coli* strain CFT073 and selected clinical isolates. Additionally, biofilm quantification was performed by spectrophotometry. We observed that clinical isolates Uro 547, Uro 553, Uro 649, Uro 678, and Uro 688 showed a higher extent on biofilm formation compared to *E. coli* strain CFT073, while a lesser extent of biofilm formation was observed in clinical isolates Uro 565, Uro 566, Uro 611, Uro 676, and Uro 692 (Figure 2(b)). Biofilm formation was undetectable only in clinical isolate Uro 567. In addition, to determine whether there was any effect of bacterial growth kinetics on biofilm formation, clinical isolates that formed equal or more biofilm than *E. coli* strain CFT073 (Figure 3(a)), as well as strains that formed little or no biofilm (Figure 3(b)), were analyzed separately. A similar pattern in bacterial growth kinetics was found for both clinical isolates that formed equal or more biofilm than *E. coli* CFT073 (Uro 553, Uro 688, Uro 678, Uro 547, Uro 649, and Uro 572) and those clinical isolates that formed little or no biofilm (Uro 611, Uro 692, Uro 567, Uro 566, Uro 565, and Uro 676). However, regardless of the biofilm-forming capacity of all clinical isolates, we observed lower bacterial growth in stationary phase compared to *E. coli* strain CFT073 (Figures 3(a) and 3(b)). Moreover, when we compared the kinetics of bacterial growth between Uro 688 (the clinical isolate with higher biofilm formation) versus Uro 567 (nonbiofilm forming) no differences were found (Figure 3(c)).

**Figure 4:**
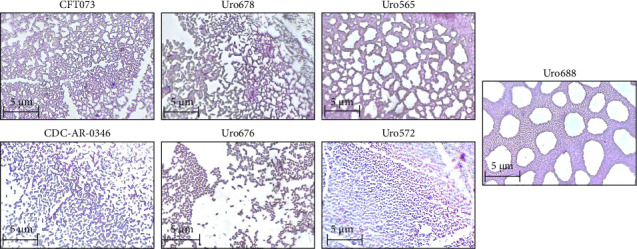
Determination of the microscopic structure of the biofilm. The microscopic structure of the biofilm of some clinical isolates was analyzed by conventional microscopy and recorded on an image analyzer, as indicated in material and methods.

On the other hand, to determine if there were differences in the microscopic structures of biofilm among the different clinical isolates, the microscopic structures of the biofilm of two clinical isolates that formed more biofilm than CFT073 (Uro 678 and Uro 688) and three clinical isolates that formed little biofilm (Uro 665, Uro 572, and Uro 676) were analyzed by conventional microscopy. The microscopic structures of biofilm of both *E. coli* strains CDC-AR-0346 and CFT073 were also analyzed. Notable differences were found in form and structural biofilm organization. Interestingly, in both clinical isolates Uro 678 and Uro 565 a network-like structure like *E. coli* strain CFT073 was observed (top), while in clinical isolates Uro 676 and Uro 572 a stacked shape like CDC-AR-0346 was observed (bottom), separations between biofilm-forming bacteria were also detected (Figure 4). In Uro 688, a combination of both microscopic biofilm structures was observed (right figure).

### 3.4. Detection of *fimH, ihf, upaB*, and *upaH* Genes in Clinical Isolates

**Figure 5:**
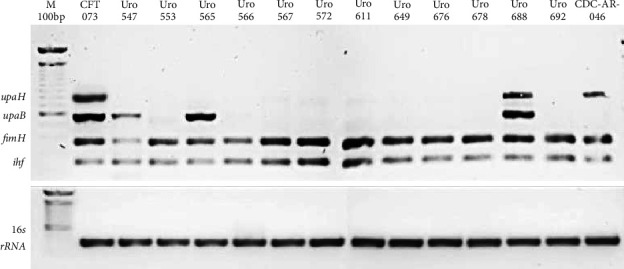
Detection of *fimH, ihf, upaB*, and *upaH* genes by a conventional PCR assay. Total DNA was purified from all samples and amplification of the *fimH, ihf, upaB*, and *upaH* genes was performed by PCR. The figure shows the PCR amplification products from all samples, subjected to electrophoresis on 1% agarose gel, stained with SYBR green and visualized on the iBright CL1500 imaging system. Clinical isolates were identified according to clinical history number.

We determined whether the *fimH, ihf, upaB*, and *upaH loci* were resent in all clinical isolates the corresponding proteins have been associated with biofilm formation in UPEC strains. As expected, *E. coli* strain CFT073 contained all four *loci* as did Uro 688 (Figure 5). Interestingly, all clinical isolates contained the *fimH* and *ihf* genes, while *upaB* was present in only three of the 12 isolates. In contrast, multidrug-resistant *E. coli* strain CDC-AR-0346 strain contained the *fimH, ihf*, and *upaH* genes, but lacked *upaB*.

### 3.5. Expression of *fimH*, *ihf*, *upaB*, and *upaH* Genes in Suspension-Grown Bacteria Versus Biofilm

**Figure 6:**
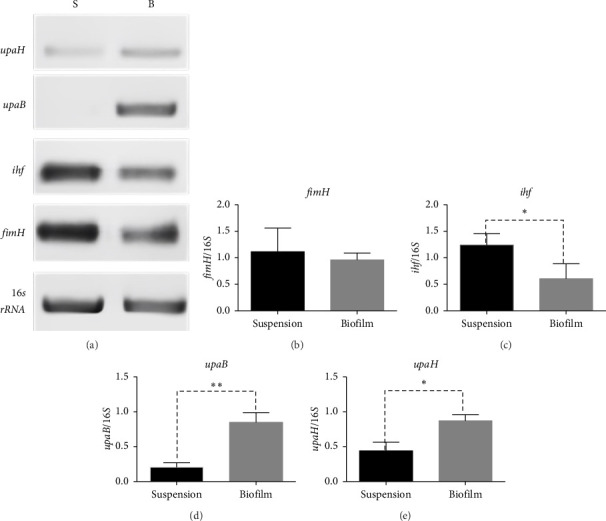
RNA expression levels of *fimH, ihf, upaB*, and *upaH* genes of *E. coli* strain CFT073 in biofilm and suspension grown bacteria. Total RNA was purified from biofilm and bacteria in suspension from the same well and differential mRNA expression was determined by RT-PCR, using oligonucleotides specific for *upaB, upaH, fimH*, and *ihf* genes, as indicated in material and methods. (a) Shows the mRNA expression levels of *fimH, ihf, upaB*, and *upaH* in biofilm (B) and suspension-grown bacteria (S) of *E. coli* strain CFT073. RT-PCR products were subjected to 1% agarose gel electrophoresis, stained with SYBR green, and visualized on the iBright CL1500 imaging system. Expression of the *16s rRNA* subunit was used as a control. The bar graphs represent the mRNA fold change of the *fimH* (b), *ihf* (c), *upaB* (d), and *upaH* (e) genes relative to *16S rRNA* subunit in both biofilm (gray bars) and suspension grown bacteria (black bars). Each bar represents the mean ± SD of each sample quantified by triplicate. ⁣^∗^*p* < 0.05, ⁣^∗∗^*p* < 0.01.

In the reference *E. coli* strain CFT073, the *fimH, ihf, upaB*, and *upaH* transcript showed different responses to growth conditions: *upaB* and *upaH* were upregulated in biofilm (B) compared with suspension growth (S), whereas *fimH* and *ihf* were slightly downregulated in B (Figure 6(a)). Interestingly, *upaB* was observed in biofilm, but not in bacteria grown in suspension. For bacteria grown in suspension and biofilm no significant differences in *fimH* expression were found (Figure 6(b)). However, *ihf* expression was higher in suspension than in biofilm (Figure 6(c)). Whereas the biofilm showed higher expression levels of both *upaB* (Figure 6(d)) and *upaH* (Figure 6(e)) compared to suspension.

**Figure 7:**
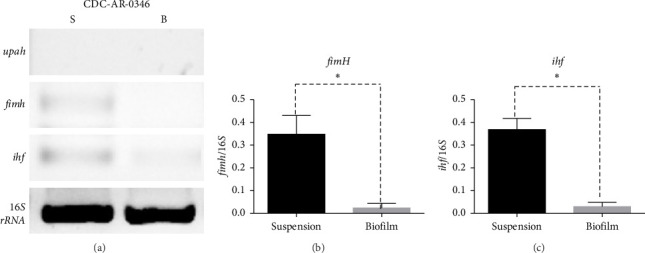
RNA expression levels of *upaH, fimH*, and *ihf* and genes of multidrug-resistant *E. coli* strain CDC-AR-0346 in biofilm and suspension grown bacteria. (a) Shows the mRNA expression levels of *upaH, fimH*, and *ihf* in biofilm (B) and suspension-grown bacteria (S) of multidrug-resistant *E. coli* strain CDC-AR-0346. Expression of the *16s rRNA* subunit was used as a control. The bar graphs represent the mRNA fold change of the *fimH* (b) and *ihf* (c) relative to *16s rRNA* subunit in both biofilm (gray bars) and suspension grown bacteria (black bars). Each bar represents the mean ± SD of each sample quantified by triplicate. ⁣^∗^*p* < 0.05, ⁣^∗∗^*p* < 0.01.

Moreover, the expression of *fimH, ihf*, and *upaH* genes was also evaluated in the multidrug-resistant *E. coli* strain CDC-AR-0346. Interestingly, it was found that both *fimH* (Figures 7(a) and 7(b)) and *ihf* (Figures 7(a) and 7(c)) were expressed in suspension, but not in biofilm, while the expression of *upaH* was not detected in bacteria grown in suspension nor in biofilm (Figure 7(a)). In addition, the expression of *fimH, ihf, upaB*, and *upaH* genes were evaluated in bacteria grown in suspension and in biofilm of three clinical isolates, with different capacities to form biofilms. The clinical isolates Uro 688 (in which 4 genes were detected), Uro 565 (3 genes detected), and Uro 572 (2 genes detected).

**Figure 8:**
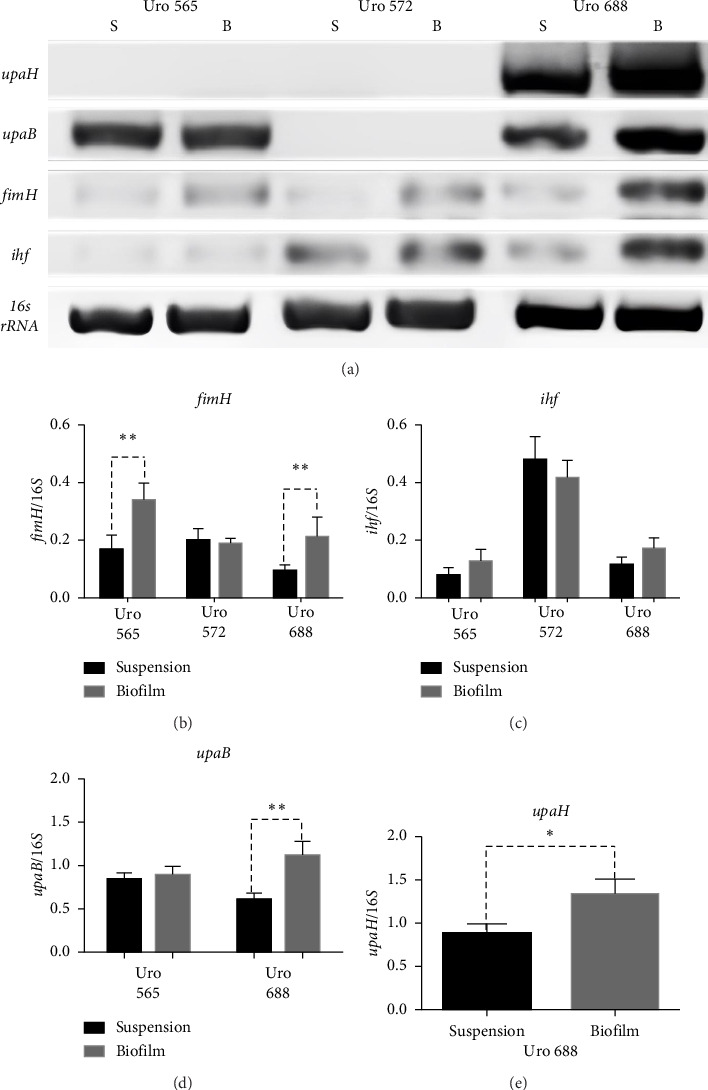
RNA expression levels of *fimH, ihf, upaB*, and *upaH* genes of *E. coli* strain from clinical isolates in biofilm and suspension-grown bacteria. (a) Shows an agarose gel with the mRNA expression levels of *fimH, ihf, upaB*, and *upaH* in biofilms (B) and suspension-grown bacteria (S) of Uro 565 (a strong biofilm-forming strain), Uro572 (a moderate biofilm-forming strain), and Uro688 (a weak biofilm-forming strain). Expression of the *16s rRNA* subunit was used as a control. The bar graphs represent the mRNA fold change of the fimH (b), ihf (c), upaB (d), and upaH (e) genes relative to 16s rRNA subunit in both biofilm (gray bars) and suspension-grown bacteria (black bars). Each bar represents the mean ± SD of each sample quantified by triplicate. ⁣^∗^*p* < 0.05, ⁣^∗∗^*p* < 0.01.

Figure 8(a) shows a representative image of the RNA levels of four genes detected in suspension-grown (S) and biofilm (B) bacteria by electrophoresis assay. Thus, in Uro 565 and Uro 688 the expression levels of *fimH* were higher in biofilm than in suspension, while the expression levels of *fimH* were similar in Uro 572 in both suspension and biofilm (Figure 8(b)). Similarly, there was no difference in *ihf* expression from bacteria grown in suspension versus those from biofilm in the three clinical isolates, although *ihf* was expressed at higher levels in Uro 572 than in Uro 565 and Uro 688 (Figure 8(c)).

Regarding the expression of *upaB*, in Uro 565 no differences were observed between the bacteria grown in suspension and biofilm; while in Uro 688 showed a higher level of *upaB* expression in biofilm compared to suspension (Figure 8(d)). Finally, the *upaH* gene was detected only in Uro 688, where *upaH* expression was higher in biofilm than in suspension-grown bacteria (Figure 8(e)).

## 4. Discussion

*Escherichia coli* is a bacterial species frequently implicated in UTIs, causing more severe relapses and chronic infections. The formation of biofilms confers to bacteria the capacity to evade the host's immune system and increases resistance to antibiotic treatment [19]. In the United States, the National Institutes of Health has reported that about 80% of infections in humans are related to biofilms-forming bacteria [20]. While in Mexico, it was reported that 30% of patients with UTI by *E. coli* showed resistance to antibiotics [21]. It is important to mention that worldwide, *E. coli* from clinical isolates has typically shown increased resistance to ampicillin and tetracycline [22, 23]. Here, we have shown preliminary data on the differential expression of four genes associated with biofilm-forming UPECs, (*fimH, ihf, upaB* and *upaH*), in both bacteria grown in suspension and biofilm. The *E. coli* strain UPEC CFT073, the multidrug-resistant *E. coli* strain CDC-AR-0346, as well as clinical isolates from patients with UTI were used. To date, the genes and regulatory mechanisms of biofilm formation by UPEC strains have not been fully described.

Importantly, these four genes are considered virulence factors in many *E. coli* strains and play an important role in biofilm formation and antibiotic resistance. The *fim*H gene is highly conserved and has been commonly detected among *E. coli* isolates. The product of this gene is the *fimH* protein that constitutes Type I fimbriae, which mediates early adhesion of UPEC to urothelial cells, an important step in bacterial colonization and bacterial biofilm formation [24]. The *ihf* is a nucleotide-associated protein, and its expression is dependent on bacterial growth rate. It can function as an activator or suppressor in the transition from motile cells to biofilm formation [25]. It is detected by neighboring bacteria as a signaling protein that induces and promotes the complex community structure of biofilm during the early stages of bacterial growth [26]. To date, several UPEC AT proteins associated with virulence and biofilm formation have been characterized, including both surface-localized *upaH* and *upaB* [16]. Also, *upaB*, and *upaH* contribute to early colonization of the mouse bladder in the murine UTI model, increasing adhesion, invasion, and biofilm formation. Although UPEC strains possess multiple AT-encoding genes, very little is known about the function of their produced proteins [15]. Many reports have studied the regulation of gene expression and biofilm formation after several days of bacterial culture. Here, bacteria were studied in 24-h cultures, in which bacteria stop swimming freely and initiate biofilm formation to protect themselves from harmful substances in the microenvironment and, in some cases, acquire antibiotic resistance. In this regard, it is widely known that during the transition from suspension growth to biofilm, there is a regulation of gene expression during the different stages of bacterial growth [27].

On the other hand, it has been described that human diseases and colonization of medical devices are related to pathogenic microorganisms growing on biofilms and these microorganisms are highly resistant to antimicrobial treatments. It has previously been described that biofilm formation may be involved in antibiotic resistance [6]. Also, it has been found that 50%–80% of *E. coli* strains from patients with UTI are biofilm producers [6, 22, 28]. As for antibiotic resistance, we observed that most *E. coli* strains from clinical isolates were resistant to at least one antibiotic, but not to nitro derivatives. It is widely known that in *E. coli* and many other bacteria strains, an important mechanism of antibiotic resistance is the expression and activity of enzymes called beta-lactamases [29, 30]. Genes encoding for beta-lactamases are often located into plasmids, which can be transferred from bacterium to bacterium [31]. Here, most of *E. coli* strains showed antibiotic resistance, but no plasmids were found in the clinical isolates Uro 547, Uro 553, Uro 565, Uro 649, Uro 676, and Uro 678. However, antibiotic resistance genes can also be integrated into the bacterial genome. Therefore, we cannot rule out that in the plasmid-negative samples, antibiotic resistance genes could be integrated into the bacterial genome [32]. Importantly, several bacterial strains contain megaplasmids that confer antibiotic resistance, and these are not detected by standard electrophoresis techniques. Further exploration of plasmid mapping through pulsed-field gel electrophoresis could be used in the future to improve the understanding of these processes.

The biofilm formation ability of bacteria is commonly classified as strong, moderate, and weak [33, 34]. In this study, to obtain accurate results on biofilm formation, *E. coli* strain CFT073 was used as a control to determine the difference in biofilm formation between clinical isolates. Biofilm formation has been reported to be dependent on bacterial growth conditions [35, 36]. Furthermore, for bacteria such as *E. coli*, biofilm formation has been reported to begin between 4 and 8 h after culture initiation and peak at 24 h [37]. For this reason, it was important to determine whether bacterial growth of the clinical isolates used in this study would have an effect on biofilm formation. Although optimal and controlled culture conditions were used here, significant differences in the capability to form biofilms were found. It is important to mention that the same pattern of bacterial growth was observed between the clinical isolate Uro 688 (which showed greater biofilm formation) and Uro 567 (in which no biofilm was formed). However, slightly lower bacterial growth was observed for clinical isolates Uro 688 and Uro 567 compared to *E. coli* strain CFT073. Therefore, we have shown that capability to form biofilms is independent of bacterial growth at least in vitro. Likewise, the type of biofilm structure in clinical isolates was microscopically analyzed, two predominant types were found: a network type like *E. coli* strain CFT073, and another structure in which there is a stacking of bacteria similar to enteroaggregative *E. coli* strains [38]. Interestingly enteroaggregative *E. coli* are pathogenic strains, also termed EAEC/UPEC hybrids that have been associated with UTIs [39, 40]. Additionally, most pathogenic *E. coli* strains, including UPEC belong to phylogenetic Group B2, which is the predominant group of bacteria in clinical isolates from patients with UTI [23, 28]. It is well known that both EAEC and EAEC/UPEC hybrid strains are associated with phylogenetic groups A, B1, B2, and D [41–43]. Here, differences in the type of microscopic biofilm structures were found in clinical isolate samples, but these biofilm structures are quite similar to those found in Group B strains, suggesting a phylogenetic relationship. However, it is important to evaluate the changes in biofilm architecture over time to determine if the structural differences persist in the reference and clinical isolates.

Previously, several genes associated with biofilm formation have been detected in *E. coli* from clinical isolates of patients with UTI, including: *fimA, fimH, papAH, papC, papEF*, and *mrkABCDF* [44–46]. Moreover, other genes related to multidrug resistance *E. coli* strains such as *fimH* and *ompT* have also been detected [47]. However, genes encoding the AT proteins *upaB* and *upaH*, as well as *ihf* gene, have been poorly studied in clinical isolates. Although it is widely known that deletion of these genes in *E. coli* strain CFT073 decreased the ability to form biofilms [14–16]. Here it was found that *fimH* and *ihf* genes were expressed in all clinical isolate samples, regardless of both antibiotic resistance and biofilm-forming ability, while *upaH* and/or *upaB* genes were detected in a few clinical isolate samples. Interestingly, expression of *fimH, ihf, upaB*, and *upaH* genes was found only in Uro 688, which formed the largest biofilm (similar to CFT073). However, previous studies have shown no differences in the genes associated with Type 1 and P fimbriae (*fimA, fimH, papEF,* and *papC*), as well as in the F1C fimbriae gene (*foc/G*) among different biofilm-producing UPEC strains, but in this study only biofilm bacteria and not suspension-grown bacteria were used [44]. In contrast, plasmids were not found in *E. coli* strain CFT073, but the presence *of fimH, ihf, upaB*, and *upaH* genes was detected. In addition, a significant increase in *upaH* and *upaB* mRNA levels was identified in the biofilm with respect to suspension-grown bacteria, while *ihf* mRNA levels decreased in the biofilm. This suggests that biofilm formation could be regulated by mechanisms dependent on differential gene expression of *upaH, upaB*, and *ihf*. With regard to *fimH* mRNA levels, no differences were found between biofilm and suspension grown bacteria, suggesting that it does not play an important role in biofilm formation. Interestingly, a decrease on *fimH* and *ihf* mRNA levels in the biofilm was observed in multidrug-resistant *E. coli* strain CDC-AR-0346 relative to suspension-grown bacteria, while *upaH* mRNA levels remained unchanged. Interestingly, from clinical isolates the expression of *fimH, ihf, upaB*, and *upaH* genes in the biofilm compared to suspension-grown bacteria was completely different to both CFT073 and the multidrug-resistant *E. coli* strain CDC-AR-0346. Thus, in Uro 688 higher levels of *fimH, ihf, upaB*, and *upaH* mRNA were detected in the biofilm than in suspension grown bacteria, whereas in Uro 565 only higher levels of *fimH* and *ihf* mRNA were detected in the biofilm, and in Uro 572 no differences in *fimH* or *ihf* mRNA levels were detected in the biofilm or in suspension grown bacteria. Therefore, similar to both CFT073 and the multidrug-resistant *E. coli* strain CDC-AR-0346, the biofilm-forming ability of clinical isolates could be related to mechanisms regulated by differential gene expression. On the other hand, it is very important to highlight that most of the strains did not present the *upaH* gene. It was only detected in CFT073, CDC-AR-046, and Uro 688. However, when the expression of this gene was evaluated, it was observed that *upaH* was expressed in both CFT073 (Figure 6) and Uro 688 (Figure 8), but not in CDC-AR-046 (Figure 7). Interestingly, in both CFT073 and Uro688 the expression levels were higher in the biofilm than in suspension-grown bacteria. Therefore, we can suggest that although this gene was neither detected nor expressed by most of the *E. coli* strains, it may play an important role in biofilm formation; and its expression may depend on the *E. coli* bacteria strain. Additionally, it is important to further investigate the mechanisms of *fimH, ihf, upaB*, and *upaH* gene expression regulation in the biofilm; as well as the implication of quorum sensing, because it is well known that is critical for the development and maturation of bacterial biofilms.

Additionally, in *E. coli*, it has been described that adhesion mechanisms and biofilm formation may be regulated by gene on/off systems [48], such as TosR [49], and *rfaH* [50]. In this regard, ipuS has been described to regulate the expression of *upaE*, an adhesion-associated AT [51]. However, the mechanisms of regulation of the ATs *upaB*, *upaH*, and *ihf* and their role in biofilm formation and type of microscopic biofilm structure are not well understood. An interesting finding of this work is that all *E. coli* strains of clinical isolates showed resistance to at least one antibiotic, and biofilm formation was observed in most of them. However, plasmids were detected as possible mobile elements for transferring antibiotic resistance in *E. coli* from clinical isolates Uro 566, Uro 567, Uro 572, Uro 611, Uro 688, and Uro 692, but not in samples Uro 547, Uro 553, Uro 565, Uro 649, Uro 676, and Uro 678; suggesting that antibiotic resistance genes may be integrated into the bacterial genome. Importantly, all clinical isolates had antibiotic resistance, and only the clinical isolate Uro567 did not form biofilm, so it can be speculated that biofilm formation may also play an important role in antibiotic resistance. In this regard, biofilm has been reported to possess the ability to resist adverse environmental factors, including the effect of antibiotics. Therefore, the antibiotic resistance of biofilm-forming bacterial cells could be very high with respect to planktonic cells [52]. While antibiotic resistance may be due to multiple mechanisms, including genes encoded in plasmids or into the bacterial genome [53]. Also, we observed that the genes associated with biofilm formation in UPEC *fimH* and *ihf* were detected in all samples; but *upaB* and *upaH* were only detected in a few *E. coli* samples. Furthermore, we found that these genes are differentially expressed in biofilm- and suspension-grown bacteria. It is important to mention that although this study did not show who and/or how the expression of *fimH, ihf, upaB*, and *upaH* genes is regulated, it was found that the expression of these genes is different in biofilm and in bacteria grown in suspension, which could be of great importance in determining the behavior of bacteria; as well as the mechanisms of antibiotic resistance and immune system evasion.

Finally, bacterial ability to adhere to different surfaces, as well as biofilm formation, has been associated with the virulence of UPECs. Previous studies on virulence genes and biofilm formation have shown significant inconsistencies [54–57]. In this study, no consistent results were found between gene expression with the ability in biofilm formation, nor the type of biofilm structure. An important limitation of our study was the small number of clinical isolates used, so it is necessary to evaluate a larger number of samples to obtain reliable results. Even so, we found important differences on the expression of *fimH, ihf, upaB*, and *upaH* genes between biofilm- and suspension-cultured bacteria.

Finally, it is important to emphasize that in this study we have shown preliminary data. The study shows important limitations that should be pointed out; among these are the following: the results obtained correspond to a small number of *E. coli* strains, including UPEC CFT073, *E. coli* CDC-AR-0346, and 12 clinical isolates from a single hospital, so in future experiments it will be necessary to study a more diverse selection of strains from different origins and with different antibiotic resistance profiles. In addition, to reduce the variability found in biofilm formation between clinical isolates and the reference strain CFT073, it is also important to analyze clinical isolates with a similar genetic background. Because only one of them (Uro 688) has the four genes evaluated as CFT073. Also, in this study the expression of only four genes related to biofilm formation was addressed, so for a more complete understanding of biofilm formation dynamics, it will be necessary to include a greater diversity of genes involved in biofilm formation. Posttranscriptional or post-translational regulatory mechanisms of gene expression should also be included. In addition, it would be interesting to include other potentially critical factors that contribute to the resistance of biofilms, such as quorum sensing systems or efflux pumps for better scientific scope. Even so, our preliminary findings on the differential expression of genes involved in biofilm formation could be useful for understanding bacterial virulence and pathogenesis. However, further experiments are still required to improve our understanding of these processes. Complete elucidation of these mechanisms could provide new insights that would facilitate the development of therapies and the prevention of biofilm-related UTIs. Thus, our data could provide important clues about gene regulation on biofilm formation.

## 5. Conclusions

Clearly, biofilm formation increases bacterial persistence in the urinary tract by protecting bacteria from various antibiotic treatments as well as providing an evasion mechanism for the immune system. Therefore, our preliminary findings on the differential expression of genes involved in biofilm formation could be useful to understand bacterial virulence and pathogenesis, as well as try to improve the treatment of UTIs. However, it is also important to investigate other genes and structural proteins involved in the regulation of biofilm formation. Additionally, in some bacteria epigenetic mechanisms have been reported in the formation of biofilms, so we cannot rule out this possibility and it would still be interesting to carry out studies in this regard. Furthermore, due to the inconsistencies in previously reported results, it is important to conduct further studies of genetic regulation in biofilm formation. Differences between the reported results may be due to the origin and variations of the *E. coli* strains detected, the bacterial culture media and methods used, as well as the number of UPEC clinical isolate samples collected and used in each study.

## Figures and Tables

**Table 1:** Sequence of PCR and RT-PCR primers.

Gene	GenBank access number	Primer sequence	Tm (°C)	PCR product size (bp)
*ihf*	EU903199.1	F: 5′ CTTGCCACCCAGCAATCGCA 3′	61.2	237
		R: 5′ ATCGCGCAGTTCTTTACCAGGT 3′	59.0	

*fimH*	GQ487191.1	F: 5′ CGCCAATGGTACCGCAATCCCTA 3′	61.5	370
		R: 5′ CACGGCAATTAATGAGCCAGCT 3′	58.8	

*upaB*	KP729058.1	F: 5′ CCATCTACTCTGATGTCCTGGCCC 3′	60.7	494
		R: 5′ GCCTGTGGCTTTTAGAGTGCTGTC 3′	60.2	

*upaH*	FJ719778.1	F: 5′ AGCGTTGATGGGAACTCATC 3′	58.0	680
		R: 5′ GGTAACGCAGTGGTATCGTT 3′	58.0	

*16S rRNA*	X80724.1	F: 5′ TCCTACGGGAGGCAGCAG T 3′	60.8	466
		R: 5′ GACTACCAGGGTATCTAATCCTGTT 3′	57.2	

Abbreviations: F = forward, R = reverse.

**Table 2:** Test for resistance to antibiotics of *E. coli* strains.

Antibiotic	CFT073	Uro 547	Uro 553	Uro 565	Uro 566	Uro 567	Uro 572	Uro 611	Uro 649	Uro 676	Uro 678	Uro 688	Uro 692
*β* lactams	−	+	+	−	+	−	−	+	−	−	+	+	−
Tetracyclines	−	+	+	−	+	+	+	+	−	+	+	−	−
Nitro derivatives	−	+	−	−	−	−	−	−	−	−	−	−	−
Aminoglycosides	−	−	−	−	+	+	−	−	−	−	−	−	−
Sulfonamides and diaminopyridines	−	+	+	−	−	−	−	+	−	−	+	−	−
Quinolones	−	+	+	−	+	−	−	−	−	−	+	−	+

*Note:* Antibiogram tests were performed as indicated in the materials and methods section. Each row represents the antibiotic, and each column represents an *E. coli* sample. The minus sign indicates no bacterial growth (antibiotic susceptibility), and the plus sign indicates bacterial growth (antibiotic resistance). Clinical isolates were classified according to the clinical history number. The *E. coli* strain CFT073 was used as a negative control. *β* lactams: ampicillin, amoxicillin-clavulanate, piperacillin-tazobactam, cefuroxime, cefoxitin, ceftazidime, ceftriaxone, cefepime, ertapenem, imipenem, meropenem tetracyclines: tetracycline; nitro derivatives: nitrofurantoin. Aminoglycosides: amikacin, gentamicin; sulfonamides and diaminopyridines: trimethoprim-sulfamethoxazole; quinolones: ciprofloxacin, levofloxacin.

## Data Availability

The data presented in this study are available on request from the corresponding author.

## Supporting Information

Supporting Table 1 shows the full antibiogram of all clinical isolates used in this study.

## References

[1] Foxman B. (2010). The Epidemiology of Urinary Tract Infection. *Nature Reviews Urology*.

[2] Waller T. A., Pantin S. A. L., Yenior A. L., Pujalte G. G. A. (2018). Urinary Tract Infection Antibiotic Resistance in the United States. *Primary Care: Clinics in Office Practice*.

[3] Busch R., Huland H. (1984). Correlation of Symptoms and Results Direct Bacterial Localization Patients with Urinary Tract Infections. *The Journal of Urology*.

[4] Paterson D. L., Bonomo R. A. (2005). Extended-Spectrum *β*-Lactamases: A Clinical Update. *Clinical Microbiology Reviews*.

[5] Shafrin J., Marijam A., Joshi A. V. (2022). Economic Burden of Antibiotic-Not-Susceptible Isolates in Uncomplicated Urinary Tract Infection: Analysis of a US Integrated Delivery Network Database. *Antimicrobial Resistance and Infection Control*.

[6] Behzadi P., Urbán E., Gajdács M. (2020). Association Between Biofilm-Production and Antibiotic Resistance in Uropathogenic *Escherichia coli* (UPEC): An In Vitro Study. *Diseases*.

[7] Flores-Mireles A., Hreha T. N., Hunstad D. A. (2019). Pathophysiology, Treatment, and Prevention of Catheter-Associated Urinary Tract Infection. *Topics in Spinal Cord Injury Rehabilitation*.

[8] Flemming H. C., Wingender J., Szewzyk U., Steinberg P., Rice S. A., Kjelleberg S. (2016). Biofilms: An Emergent Form of Bacterial Life. *Nature Reviews Microbiology*.

[9] Stewart P. S., Franklin M. J. (2008). Physiological Heterogeneity in Biofilms. *Nature Reviews Microbiology*.

[10] Kotakonda Arunasri S. V. M. (2019). Biofilms: Microbial Life on the Electrode Surface. *Biomass, Biofuels and Biochemicals, Microbial Electrochemical Technology2019*.

[11] Behzadi P. (2020). Classical Chaperone-Usher (CU) Adhesive Fimbriome: Uropathogenic *Escherichia coli* (UPEC) and Urinary Tract Infections (UTIs). *Folia Microbiologica*.

[12] Schembri M. A., Christiansen G., Klemm P. (2001). FimH-Mediated Autoaggregation of *Escherichia coli*. *Molecular Microbiology*.

[13] Foroogh N., Rezvan M., Ahmad K., Mahmood S. (2021). Structural and Functional Characterization of the FimH Adhesin of Uropathogenic *Escherichia coli* and Its Novel Applications. *Microbial Pathogenesis*.

[14] Devaraj A., Justice S. S., Bakaletz L. O., Goodman S. D. (2015). DNABII Proteins Play a Central Role in UPEC Biofilm Structure. *Molecular Microbiology*.

[15] Allsopp L. P., Beloin C., Ulett G. C. (2012). Molecular Characterization of UpaB and UpaC, Two New Autotransporter Proteins of Uropathogenic *Escherichia coli* CFT073. *Infection and Immunity*.

[16] Allsopp L. P., Totsika M., Tree J. J. (2010). UpaH Is a Newly Identified Autotransporter Protein that Contributes to Biofilm Formation and Bladder Colonization by Uropathogenic *Escherichia coli* CFT073. *Infection and Immunity*.

[17] Green M. R., Sambrook J. (2016). Preparation of Plasmid DNA by Alkaline Lysis With Sodium Dodecyl Sulfate: Minipreps. *Cold Spring Harbour Protocols*.

[18] Rozwandowicz M., Brouwer M. S. M., Fischer J. (2018). Plasmids Carrying Antimicrobial Resistance Genes in Enterobacteriaceae. *Journal of Antimicrobial Chemotherapy*.

[19] Ballén V., Cepas V., Ratia C., Gabasa Y., Soto S. M. (2022). Clinical *Escherichia coli*: From Biofilm Formation to New Antibiofilm Strategies. *Microorganisms*.

[20] Davies D. (2003). Understanding Biofilm Resistance to Antibacterial Agents. *Nature Reviews Drug Discovery*.

[21] Molina-López J., Aparicio-Ozores G., Ribas-Aparicio R. M. (2011). Drug Resistance, Serotypes, and Phylogenetic Groups Among Uropathogenic *Escherichia coli* Including O25-ST131 in Mexico City. *J Infect Dev Ctries*.

[22] Zhao F., Yang H., Bi D., Khaledi A., Qiao M. (2020). A Systematic Review and Meta-Analysis of Antibiotic Resistance Patterns, and the Correlation between Biofilm Formation With Virulence Factors in Uropathogenic *E. coli* Isolated from Urinary Tract Infections. *Microbial Pathogenesis*.

[23] De Souza G. M., Neto E. R. D. S., Silva A. M. d. (2019). *Escherichia coli*. *Infection and Drug Resistance*.

[24] Wiles T. J., Kulesus R. R., Mulvey M. A. (2008). Origins and Virulence Mechanisms of Uropathogenic *Escherichia coli*. *Experimental and Molecular Pathology*.

[25] McLeod S. M., Johnson R. C. (2001). Control of Transcription by Nucleoid Proteins. *Current Opinion in Microbiology*.

[26] Srivastava S., Bhargava A. (2016). Biofilms and Human Health. *Biotechnology Letters*.

[27] Beloin C., Roux A., Ghigo J. M. (2008). *Escherichia coli* Biofilms. *Current Topics in Microbiology and Immunology*.

[28] Javed S., Mirani Z. A., Pirzada Z. A. (2021). Phylogenetic Group B2 Expressed Significant Biofilm Formation Among Drug Resistant Uropathogenic *Escherichia coli*. *Libyan Journal of Medicine*.

[29] Bush K. (2018). Past and Present Perspectives on *β*-Lactamases. *Antimicrobial Agents and Chemotherapy*.

[30] Poirel L., Madec J. Y., Lupo A. (2018). Antimicrobial Resistance in *Escherichia coli*. *Microbiology Spectrum*.

[31] Bush K., Bradford P. A. (2020). Epidemiology of *β*-Lactamase-Producing Pathogens. *Clinical Microbiology Reviews*.

[32] Gomi R., Yamamoto M., Tanaka M., Matsumura Y. (2022). Chromosomal Integration of blaCTX-M Genes in Diverse *Escherichia coli* Isolates Recovered From River Water in Japan. *Current Research in Microbial Sciences*.

[33] Boroumand M., Sharifi A., Ghatei M., Sadrinasab M. A., Sadrinasab M. (2021). Evaluation of Biofilm Formation and Virulence Genes and Association With Antibiotic Resistance Patterns of Uropathogenic *Escherichia coli* Strains in Southwestern Iran. *Jundishapur Journal of Microbiology*.

[34] Ponnusamy P., Natarajan V., Sevanan M. (2012). In Vitro Biofilm Formation by Uropathogenic *Escherichia coli* and Their Antimicrobial Susceptibility Pattern. *Asian Pacific Journal of Tropical Medicine*.

[35] Adamus-Białek W., Kubiak A., Czerwonka G. (2015). Analysis of Uropathogenic *Escherichia coli* Biofilm Formation under Different Growth Conditions. *Acta Biochimica Polonica*.

[36] Eberly A. R., Floyd K. A., Beebout C. J. (2017). Biofilm Formation by Uropathogenic *Escherichia coli* is Favored Under Oxygen Conditions That Mimic the Bladder Environment. *International Journal of Molecular Sciences*.

[37] Colón-González M., Méndez-Ortiz M. M., Membrillo-Hernández J. (2004). Anaerobic Growth Does Not Support Biofilm Formation in *Escherichia coli* K-12. *Research in Microbiology*.

[38] Jenkins C. (2018). Enteroaggregative *Escherichia coli*. *Current Topics in Microbiology and Immunology*.

[39] Lara F. B., Nery D. R., de Oliveira P. M. (2017). Virulence Markers and Phylogenetic Analysis of *Escherichia coli* Strains with Hybrid EAEC/UPEC Genotypes Recovered From Sporadic Cases of Extraintestinal Infections. *Frontiers in Microbiology*.

[40] Nascimento J. A. S., Santos F. F., Valiatti T. B. (2021). Frequency and Diversity of Hybrid *Escherichia coli* Strains Isolated From Urinary Tract Infections. *Microorganisms*.

[41] Imuta N., Ooka T., Seto K. (2016). Phylogenetic Analysis of Enteroaggregative *Escherichia coli* (EAEC) Isolates From Japan Reveals Emergence of CTX-M-14-Producing EAEC O25:H4 Clones Related to Sequence Type 131. *Journal of Clinical Microbiology*.

[42] Guiral E., Mendez-Arancibia E., Soto S. M. (2011). CTX-M-15-producing Enteroaggregative *Escherichia coli* as Cause of Travelers’ Diarrhea. *Emerging Infectious Diseases*.

[43] Denamur E., Clermont O., Bonacorsi S., Gordon D. (2021). The Population Genetics of Pathogenic *Escherichia coli*. *Nature Reviews Microbiology*.

[44] Zamani H., Salehzadeh A. (2018). Biofilm Formation in Uropathogenic *Escherichia coli*: Association With Adhesion Factor Genes. *Turkish Journal of Medical Sciences*.

[45] Ong C. L., Ulett G. C., Mabbett A. N. (2008). Identification of Type 3 Fimbriae in Uropathogenic *Escherichia coli* Reveals a Role in Biofilm Formation. *Journal of Bacteriology*.

[46] Schembri M. A., Kjaergaard K., Klemm P. (2003). Global Gene Expression in *Escherichia coli* Biofilms. *Molecular Microbiology*.

[47] Baldiris-Avila R., Montes-Robledo A., Buelvas-Montes Y. (2020). Phylogenetic Classification, Biofilm-Forming Capacity, Virulence Factors, and Antimicrobial Resistance in Uropathogenic *Escherichia coli* (UPEC). *Current Microbiology*.

[48] Henderson I. R., Owen P., Nataro J. P. (1999). Molecular Switches--the on and off of Bacterial Phase Variation. *Molecular Microbiology*.

[49] Luterbach C. L., Forsyth V. S., Engstrom M. D., Mobley H. L. T. (2018). TosR-Mediated Regulation of Adhesins and Biofilm Formation in Uropathogenic *Escherichia coli*. *mSphere*.

[50] Beloin C., Michaelis K., Lindner K. (2006). The Transcriptional Antiterminator RfaH Represses Biofilm Formation in *Escherichia coli*. *Journal of Bacteriology*.

[51] Battaglioli E. J., Goh K. G. K., Atruktsang T. S., Schwartz K., Schembri M. A., Welch R. A. (2018). Identification and Characterization of a Phase-Variable Element That Regulates the Autotransporter UpaE in Uropathogenic *Escherichia coli*. *mBio*.

[52] Khan F., Pham D. T. N., Oloketuyi S. F., Kim Y. M. (2020). Antibiotics Application Strategies to Control Biofilm Formation in Pathogenic Bacteria. *Current Pharmaceutical Biotechnology*.

[53] Jian Z., Zeng L., Xu T. (2021). Antibiotic Resistance Genes in Bacteria: Occurrence, Spread, and Control. *Journal of Basic Microbiology*.

[54] Cordeiro M. A., Werle C. H., Milanez G. P., Yano T. (2016). Curli Fimbria: An *Escherichia coli* Adhesin Associated With Human Cystitis. *Brazilian Journal of Microbiology*.

[55] Ejrnæs K., Stegger M., Reisner A. (2011). Characteristics of *Escherichia coli* Causing Persistence or Relapse of Urinary Tract Infections: Phylogenetic Groups, Virulence Factors and Biofilm Formation. *Virulence*.

[56] Naves P., del Prado G., Huelves L. (2008). Correlation Between Virulence Factors and In Vitro Biofilm Formation by *Escherichia coli* Strains. *Microbial Pathogenesis*.

[57] Kanamaru S., Kurazono H., Terai A. (2006). Increased Biofilm Formation in *Escherichia coli* Isolated From Acute Prostatitis. *International Journal of Antimicrobial Agents*.

