# Supplementary material for: Differential Expression of fimH, ihf, upaB, and upaH Genes in Biofilms- and Suspension-Grown Bacteria From Samples of Different Uropathogenic Strains of Escherichia coli
Source: Int J Microbiol. 2024 Dec 12;2024:5235071. doi: 10.1155/ijm/5235071 (PMC11658850; doi:10.1155/ijm/5235071)
Supplement: Supporting Information — Additional supporting information can be found online in the Supporting Information section. [file 5235071.f1.docx]

**Suplementary Table 1.Complete antibiogram of clinical isolates of *E.coli***

| **ID** | **Antibiotic Family** | **Antibiotic** | **Resistance** |
| --- | --- | --- | --- |
| URO 547 | β lactams | Ampicillin | **+** |
|  |  | Amoxicillin-Clavulanate | **+** |
|  |  | Piperacillin-Tazobactam | - |
|  |  | Cefuroxime | **+** |
|  |  | Cefoxitin | - |
|  |  | Ceftazidime | **+** |
|  |  | Ceftriaxone | **+** |
|  |  | Cefepime | **+** |
|  |  | Ertapenem | - |
|  |  | Imipenem | - |
|  |  | Meropenem | - |
|  | Tetracyclines | Tetracycline | **+** |
|  | Nitro derivatives | Nitrofurantoin | **-** |
|  | Aminoglycosides | Amikacin | - |
|  |  | Gentamicin | **+** |
|  | Sulfonamides and diaminopyridines | Trimethoprim-Sulfamethoxazole | **+** |
|  | Quinolones | Ciprofloxacin | **+** |
|  |  | Levofloxacin | **+** |
| URO 553 | β lactams | Ampicillin | **+** |
|  |  | Amoxicillin-Clavulanate | - |
|  |  | Piperacillin-Tazobactam | - |
|  |  | Cefuroxime | - |
|  |  | Cefoxitin | - |
|  |  | Ceftazidime | - |
|  |  | Ceftriaxone | - |
|  |  | Cefepime | - |
|  |  | Ertapenem | - |
|  |  | Imipenem | - |
|  |  | Meropenem | - |
|  | Tetracyclines | Tetracycline | **+** |
|  | Nitro derivatives | Nitrofurantoin | - |
|  | Aminoglycosides | Amikacin | - |
|  |  | Gentamicin | - |
|  | Sulfonamides and diaminopyridines | Trimethoprim-Sulfamethoxazole | **+** |
|  | Quinolones | Ciprofloxacin | **+** |
|  |  | Levofloxacin | **+** |
|  |  |  |  |
|  |  |  |  |
|  |  |  |  |
|  |  |  |  |
|  |  |  |  |
|  |  |  |  |
|  |  |  |  |
|  |  |  |  |
| **ID** | **Antibiotic Family** | **Antibiotic** | **Resistance** |
| URO 565 | β lactams | Ampicillin | - |
|  |  | Amoxicillin-Clavulanate | - |
|  |  | Piperacillin-Tazobactam | - |
|  |  | Cefuroxime | - |
|  |  | Cefoxitin | - |
|  |  | Ceftazidime | - |
|  |  | Ceftriaxone | - |
|  |  | Cefepime | - |
|  |  | Ertapenem | - |
|  |  | Imipenem | - |
|  |  | Meropenem | - |
|  | Tetracyclines | Tetracycline | - |
|  | Nitro derivatives | Nitrofurantoin | - |
|  | Aminoglycosides | Amikacin | - |
|  |  | Gentamicin | - |
|  | Sulfonamides and diaminopyridines | Trimethoprim-Sulfamethoxazole | - |
|  | Quinolones | Ciprofloxacin | - |
|  |  | Levofloxacin | - |
| URO 566 | β lactams | Ampicillin | **+** |
|  |  | Amoxicillin-Clavulanate | **+** |
|  |  | Piperacillin-Tazobactam | **-** |
|  |  | Cefuroxime | **+** |
|  |  | Cefoxitin | - |
|  |  | Ceftazidime | **+** |
|  |  | Ceftriaxone | **+** |
|  |  | Cefepime | **+** |
|  |  | Ertapenem | - |
|  |  | Imipenem | - |
|  |  | Meropenem | - |
|  | Tetracyclines | Tetracycline | **+** |
|  | Nitro derivatives | Nitrofurantoin | - |
|  | Aminoglycosides | Amikacin | - |
|  |  | Gentamicin | **+** |
|  | Sulfonamides and diaminopyridines | Trimethoprim-Sulfamethoxazole | - |
|  | Quinolones | Ciprofloxacin | **+** |
|  |  | Levofloxacin | **+** |
|  |  |  |  |
|  |  |  |  |
|  |  |  |  |
|  |  |  |  |
|  |  |  |  |
|  |  |  |  |
|  |  |  |  |
|  |  |  |  |
|  |  |  |  |
| **ID** | **Antibiotic Family** | **Antibiotic** | **Resistance** |
| URO 567 | β lactams | Ampicillin | - |
|  |  | Amoxicillin-Clavulanate | - |
|  |  | Piperacillin-Tazobactam | - |
|  |  | Cefuroxime | - |
|  |  | Cefoxitin | - |
|  |  | Ceftazidime | - |
|  |  | Ceftriaxone | - |
|  |  | Cefepime | - |
|  |  | Ertapenem | - |
|  |  | Imipenem | - |
|  |  | Meropenem | - |
|  | Tetracyclines | Tetracycline | **+** |
|  | Nitro derivatives | Nitrofurantoin | - |
|  | Aminoglycosides | Amikacin | - |
|  |  | Gentamicin | **+** |
|  | Sulfonamides and diaminopyridines | Trimethoprim-Sulfamethoxazole | - |
|  | Quinolones | Ciprofloxacin | - |
|  |  | Levofloxacin | - |
| URO 572 | β lactams | Ampicillin | - |
|  |  | Amoxicillin-Clavulanate | - |
|  |  | Piperacillin-Tazobactam | **+** |
|  |  | Cefuroxime | - |
|  |  | Cefoxitin | - |
|  |  | Ceftazidime | - |
|  |  | Ceftriaxone | - |
|  |  | Cefepime | - |
|  |  | Ertapenem | - |
|  |  | Imipenem | - |
|  |  | Meropenem | - |
|  | Tetracyclines | Tetracycline | - |
|  | Nitro derivatives | Nitrofurantoin | - |
|  | Aminoglycosides | Amikacin | - |
|  |  | Gentamicin | - |
|  | Sulfonamides and diaminopyridines | Trimethoprim-Sulfamethoxazole | - |
|  | Quinolones | Ciprofloxacin | - |
|  |  | Levofloxacin | - |
|  |  |  |  |
|  |  |  |  |
|  |  |  |  |
|  |  |  |  |
|  |  |  |  |
|  |  |  |  |
|  |  |  |  |
|  |  |  |  |
|  |  |  |  |
| **ID** | **Antibiotic Family** | **Antibiotic** | **Resistance** |
| URO 611 | β lactams | Ampicillin | **+** |
|  |  | Amoxicillin-Clavulanate | **-** |
|  |  | Piperacillin-Tazobactam | - |
|  |  | Cefuroxime | - |
|  |  | Cefoxitin | - |
|  |  | Ceftazidime | - |
|  |  | Ceftriaxone | - |
|  |  | Cefepime | - |
|  |  | Ertapenem | - |
|  |  | Imipenem | - |
|  |  | Meropenem | - |
|  | Tetracyclines | Tetracycline | **+** |
|  | Nitro derivatives | Nitrofurantoin | - |
|  | Aminoglycosides | Amikacin | - |
|  |  | Gentamicin | - |
|  | Sulfonamides and diaminopyridines | Trimethoprim-Sulfamethoxazole | **+** |
|  | Quinolones | Ciprofloxacin | - |
|  |  | Levofloxacin | - |
| URO 649 | β lactams | Ampicillin | - |
|  |  | Amoxicillin-Clavulanate | - |
|  |  | Piperacillin-Tazobactam | - |
|  |  | Cefuroxime | - |
|  |  | Cefoxitin | - |
|  |  | Ceftazidime | - |
|  |  | Ceftriaxone | - |
|  |  | Cefepime | - |
|  |  | Ertapenem | - |
|  |  | Imipenem | - |
|  |  | Meropenem | - |
|  | Tetracyclines | Tetracycline | - |
|  | Nitro derivatives | Nitrofurantoin | - |
|  | Aminoglycosides | Amikacin | - |
|  |  | Gentamicin | - |
|  | Sulfonamides and diaminopyridines | Trimethoprim-Sulfamethoxazole | - |
|  | Quinolones | Ciprofloxacin | - |
|  |  | Levofloxacin | - |
|  |  |  |  |
|  |  |  |  |
|  |  |  |  |
|  |  |  |  |
|  |  |  |  |
|  |  |  |  |
|  |  |  |  |
|  |  |  |  |
|  |  |  |  |
| **ID** | **Antibiotic Family** | **Antibiotic** | **Resistance** |
| URO 676 | β lactams | Ampicillin | - |
|  |  | Amoxicillin-Clavulanate | - |
|  |  | Piperacillin-Tazobactam | - |
|  |  | Cefuroxime | - |
|  |  | Cefoxitin | - |
|  |  | Ceftazidime | - |
|  |  | Ceftriaxone | - |
|  |  | Cefepime | - |
|  |  | Ertapenem | - |
|  |  | Imipenem | - |
|  |  | Meropenem | - |
|  | Tetracyclines | Tetracycline | **+** |
|  | Nitro derivatives | Nitrofurantoin | - |
|  | Aminoglycosides | Amikacin | - |
|  |  | Gentamicin | - |
|  | Sulfonamides and diaminopyridines | Trimethoprim-Sulfamethoxazole | - |
|  | Quinolones | Ciprofloxacin | - |
|  |  | Levofloxacin | - |
| URO 678 | β lactams | Ampicillin | **+** |
|  |  | Amoxicillin-Clavulanate | - |
|  |  | Piperacillin-Tazobactam | - |
|  |  | Cefuroxime | - |
|  |  | Cefoxitin | - |
|  |  | Ceftazidime | - |
|  |  | Ceftriaxone | - |
|  |  | Cefepime | - |
|  |  | Ertapenem | - |
|  |  | Imipenem | - |
|  |  | Meropenem | - |
|  | Tetracyclines | Tetracycline | **+** |
|  | Nitro derivatives | Nitrofurantoin | - |
|  | Aminoglycosides | Amikacin | - |
|  |  | Gentamicin | - |
|  | Sulfonamides and diaminopyridines | Trimethoprim-Sulfamethoxazole | **+** |
|  | Quinolones | Ciprofloxacin | **+** |
|  |  | Levofloxacin | **+** |
|  |  |  |  |
|  |  |  |  |
|  |  |  |  |
|  |  |  |  |
|  |  |  |  |
|  |  |  |  |
|  |  |  |  |
|  |  |  |  |
|  |  |  |  |
| **ID** | **Antibiotic Family** | **Antibiotic** | **Resistance** |
| URO 688 | β lactams | Ampicillin | **+** |
|  |  | Amoxicillin-Clavulanate | **-** |
|  |  | Piperacillin-Tazobactam | - |
|  |  | Cefuroxime | - |
|  |  | Cefoxitin | - |
|  |  | Ceftazidime | - |
|  |  | Ceftriaxone | - |
|  |  | Cefepime | - |
|  |  | Ertapenem | - |
|  |  | Imipenem | - |
|  |  | Meropenem | - |
|  | Tetracyclines | Tetracycline | - |
|  | Nitro derivatives | Nitrofurantoin | - |
|  | Aminoglycosides | Amikacin | - |
|  |  | Gentamicin | - |
|  | Sulfonamides and diaminopyridines | Trimethoprim-Sulfamethoxazole | - |
|  | Quinolones | Ciprofloxacin | - |
|  |  | Levofloxacin | - |
| URO 692 | β lactams | Ampicillin | - |
|  |  | Amoxicillin-Clavulanate | - |
|  |  | Piperacillin-Tazobactam | - |
|  |  | Cefuroxime | - |
|  |  | Cefoxitin | - |
|  |  | Ceftazidime | - |
|  |  | Ceftriaxone | - |
|  |  | Cefepime | - |
|  |  | Ertapenem | - |
|  |  | Imipenem | - |
|  |  | Meropenem | - |
|  | Tetracyclines | Tetracycline | - |
|  | Nitro derivatives | Nitrofurantoin | - |
|  | Aminoglycosides | Amikacin | - |
|  |  | Gentamicin | - |
|  | Sulfonamides and diaminopyridines | Trimethoprim-Sulfamethoxazole | - |
|  | Quinolones | Ciprofloxacin | **+** |
|  |  | Levofloxacin | **+** |
|  |  |  |  |
